# Seismic Performance of Steel Fiber Reinforced High–Strength Concrete Beam–Column Joints

**DOI:** 10.3390/ma14123235

**Published:** 2021-06-11

**Authors:** Ke Shi, Mengyue Zhang, Tao Zhang, Pengfei Li, Junpeng Zhu, Li Li

**Affiliations:** 1 Huadian Zhengzhou Mechanical Design Institute Co., Ltd., Zhengzhou 450046, China; shike@zua.edu.cn; 2 College of Water Conservancy Science and Engineering, Zhengzhou University, Zhengzhou 450001, China; 3 School of Civil Engineering and Architecture, Zhengzhou University of Aeronautics, Zhengzhou 450046, China; zhangmengyue@zua.edu.cn (M.Z.); lipf2021@163.com (P.L.); zhujunpeng@zua.edu.cn (J.Z.); 4 State Key Laboratory of Green Building Materials, China Building Materials Academy, Beijing 100024, China; drlili@nwafu.edu.cn

**Keywords:** beam–column joints, steel fiber reinforced high–strength concrete, pseudo–static test, seismic performance

## Abstract

In high–strength concrete, the reinforcement concentration will cause some problems in the beam–column joints (BCJs) due to a large amount of transverse reinforcement. Hence, the main object of this paper is to prevent the reinforcement concentration and reduce the amount of transverse reinforcement in the BCJs through the ideal usage of steel fibers and reinforced high–strength concrete. Pseudo–static tests on seven specimens were carried out to investigate and evaluate the seismic performance of beam–column joints in steel fiber reinforced high–strength concrete (SFRHC). Test variables were steel fiber volume ratio, concrete strength, the stirrup ratio in the core area, and an axial compression ratio of the column end. During the test, the hysteresis curves and failure mode were recorded. The seismic indicators, such as energy dissipation, ductility, strength, and stiffness degradation, were determined. The experimental results indicated that the failure modes of SFRHC beam–column joints mainly included the core area failure and the beam end bending failure. With the increase in stirrup ratio, volume ratio of steel fiber, and axial compression ratio in the core area, both the ductility and energy consumption of beam–column joints increased, while the opposite was true for concrete strength.

## 1. Introduction

Under the earthquake load, the beam–column joints (BCJs) of frame structures are prone to brittle shear failure due to the comprehensive action of compression, bending, and shear. As the BCJs are the key part of the frame structure, their failure will easily lead to the collapse of the whole structure. Both Chinese and American seismic codes [1,2,3] put forward the requirement of “strong joints”, that is, to improve the ductility and energy dissipation of BCJs, and further to improve the seismic performance of frame structures. To meet the requirements of “strong joints”, more shear reinforcement is usually required in practical engineering, which will lead to serious casting blockage and construction difficulties of concrete [4,5,6]. In addition, this may lead to the use of larger column and beam sections or the use of more small–diameter steel bars to meet the minimum anchorage length requirement through the joint core area, which may worsen casting blockage and construction difficulties of concrete. Therefore, how to design the beam–column joints better is a key issue.

In different designs [7,8,9,10,11,12], adding randomly distributed short fibers into the concrete matrix to improve the tensile and crack resistance is an excellent method [13,14,15,16,17,18,19]. Experiments showed that steel fiber had little contribution to compressive strength [20,21,22,23,24], but it can obviously improve the compressive behavior of stress and strain after cracking and also shows apparent toughness [25,26,27,28]. Fibers are mainly divided into synthetic substances and natural substances. Discontinuous discrete fibers are fully integrated into concrete mixture in a random direction, which has significant advantages for the performance of reinforced concrete members [29,30,31,32,33,34] and reduced reinforcement congestion to a certain extent [35,36,37]. Smarzewski [38,39] studied the positive synergistic effect of steel and polypropylene fiber in reinforced concrete deep beams to replace conventional steel reinforcement. Zhao et al. [40] emphasized the beneficial effects of steel fiber on stiffness, shear capacity, and deformation of shear critical reinforced concrete beams. In addition, steel fiber can also improve the shear strength of SFRC members [41,42]. It was also proven that deformed or hooked steel fiber with a high aspect ratio could improve crack resistance, energy dissipation, and ductility index most effectively [43,44]. Chalioris [45] studied the effect of steel fiber on the response of ordinary reinforced concrete beams under reverse load by a four–point bending test. Compared with reinforced concrete beams, the overall hysteretic response and energy absorption capacity of SFRC beams are improved. Kytinou [46] studied the hysteretic behavior of steel fiber reinforced concrete slender deep beams. The results showed that SFRC beams exhibit enhanced cyclic performance in terms of residual stiffness, bearing capacity, deformation, energy dissipation capacity, and cracking performance. Hammad [47] studied the properties of structural polypropylene and steel fiber reinforced alkali slag concrete cured at room temperature. Lehner [48] studied the effect of fiber on the reference concrete of ordinary Portland cement and self–compacting concrete and compared and evaluated the basic mechanical properties of their concrete mixtures. Issa et al. [49] and Zhu et al. [50] obtained a higher ultimate compressive strain of concrete by adding steel fibers, thus improving the ductility and flexural strength of fiber–reinforced concrete beams. Abbas et al. [6] investigated the cyclic behavior of BCJs to find that the addition of steel fibers enhanced the strength, stiffness, ductility, and energy dissipation capacity of the beam–column joints. Henager [51] studied the influence of steel fiber reinforced concrete on the seismic performance of BCJs through two full–scale beam–column joints. The experimental results showed that the corner ductility coefficient, bond strength of longitudinal bars, and ultimate bending moment of the specimens with steel fibers were improved compared with those without steel fibers. Filiatrault et al. [52] carried out experiments on steel fiber reinforced concrete side column joints under low cyclic loading. The results showed that compared with the concrete beam–column joints without fiber, the ductility coefficient of concrete beam–column joints with a high content of steel fiber was greater, and the energy dissipation capacity of the structure is stronger. Gebman [53] conducted six reinforced steel fiber reinforced concrete beam–column joints and two reinforced concrete beam–column joints. 

However, the SFRC test with insufficient steel fiber content shows that the increase in tensile strength is negligible, while the improvement of properties after cracking is limited [54]. Therefore, the critical volume fraction of steel fiber was proposed to design a high–performance fiber–reinforced concrete mixture, which achieves strain hardening under direct tension and has advanced ductility and energy absorption capacity [55,56,57], such as steel fiber high–strength reinforced concrete (SFHRC). Choi and Kim [15,16] studied the influence of different steel fiber volume fractions on the compressive strength and tensile strength of high–strength steel fiber reinforced concrete beams. Li [58] tested a series of fiber–reinforced high–strength concrete beams under static and explosive loads using impact tubes. The results show that fiber can significantly improve the ductility of high–strength reinforced concrete beams under static load. Under dynamic conditions, the application of fiber–reinforced high–strength concrete can significantly improve the blasting performance. Shannag et al. [59] found that compared with reinforced concrete beam–column joints, the dissipation capacity of reinforced steel fiber high–strength concrete beam–column joints increased by 20 times, and the stiffness degradation decreased by 2 times. Ganesan [60] analyzed the influence of steel fiber volume ratio change on the seismic performance of beam–column joints through 10 reinforced steel fiber high–strength concrete beam–column joints tests and found that ductility, energy consumption, and shear capacity of beam–column joints increased with the increase in steel fiber volume ratio.

The related research indicated that the previous studies mainly focused on the seismic performance of steel fiber reinforced concrete (SFRC), with a small part involving the overall seismic performance of SFRHC, and even less local steel fiber–reinforced high–strength concrete. Therefore, the main objective of this study is to investigate the seismic performance of BCJs experimentally by adding a certain volume of steel fibers into the joint core area, adjacent beam end, and column end and reducing the amount of transverse reinforcement in the joint core area. The effects of concrete strength, the axial compression ratio of the column top, volume ratio of steel fiber, and stirrup ratio in the core area of steel fiber reinforced concrete on structural performance are studied. The research aims to improve the seismic performance of SFRHC beam–column joints, including ductility, energy absorption capacity, strength, and stiffness degradation.

## 2. Experimental Program

### 2.1. Test Specimens and Materials

In this paper, a total of seven 1/2 scale beam–column joints were designed and tested, and the effects of concrete strength, the volume ratio of steel fiber, the axial compression ratio of the column end, and stirrup ratio in the core area were considered. The concrete mixture used was composed of ordinary Portland cement, water, well–graded natural river sand, and crushed stone aggregate. The steel fibers added to the fibrous concrete mixture are hook–ended fibers to enhance the anchoring of the fibers in the matrix. The length–to–diameter ratio of the fiber used is equal to *l_f_*/*d_f_* = 35 mm/0.55 mm = 64. The nominal yield tensile strength of the fibers was 1345 MPa. The test specimens were labeled using nomenclatures such as the specimen type and specimen number. For example, the label “BCJ1” defines the specimens as follows: the first three letters indicate the specimen type, where the prefix letter “BCJ” refers to the beam–column joints in the seam joints of steel fiber–reinforced high–strength concrete (SFRHC) beams and columns, and the following digit, “1” is the specimen number. The beam–column joints between the middle column of the plane frame and the inverted beam point were selected as the research object. In terms of SFRHC specimens, BCJ1 was the control specimen, and the others were constructed with representative characteristics. Specimen BCJ0 was cast using normal concrete for comparison. The more detailed information for all specimens is listed in Table 1.

**Figure 1:**
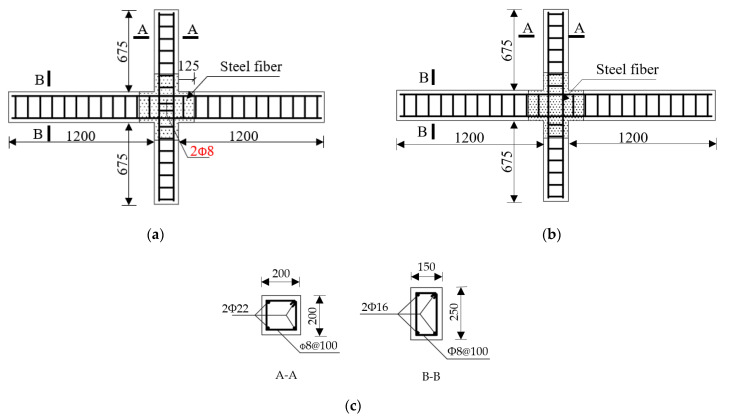
Specimen dimension and reinforcement (except for BCJ0 where no steel fiber is used): (**a**) BCJ0, BCJ1, BCJ2, and BCJ3 (the core area is equipped with stirrups); (**b**) BCJ4, BCJ5, and BCJ6 (the core area is not equipped with stirrups); (**c**) Reinforcement of specimen (unit: mm).

The detailed dimension of the specimens is shown in Figure 1. The ends of columns and beams were points of contra–flexure. All the columns have the same cross–section of 200 × 200 mm^2^ with a total height of 1600 mm, and the transverse beam has a cross–section of 150 × 250 mm^2^ with a total length of 2600 mm. The longitudinal reinforcements in the columns consisted of four B22 bars (a diameter of 16 mm and the grade HRB335 steel), and those for beams consisted of four B16 bars. Transverse reinforcements in columns, beams, and joint core area consisted of an 8 mm–diameter reinforcement. Table 1 gives the parameters and mechanical properties of the specimens. Table 2 summarizes the mechanical properties of steel bars measured in the test.

### 2.2. Experimental Device and Loading System

**Figure 2:**
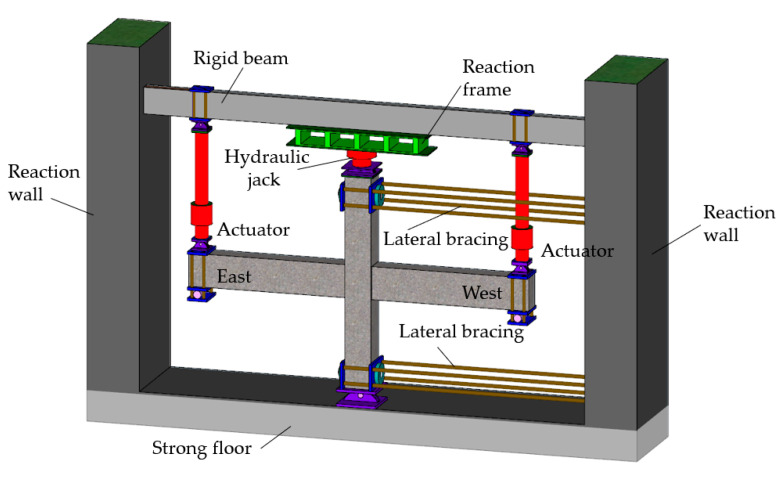
Schematic diagram.

**Figure 3:**
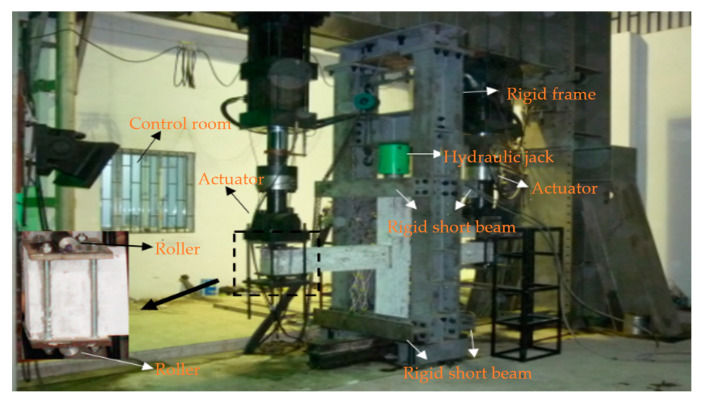
Experimental setup.

**Figure 4:**
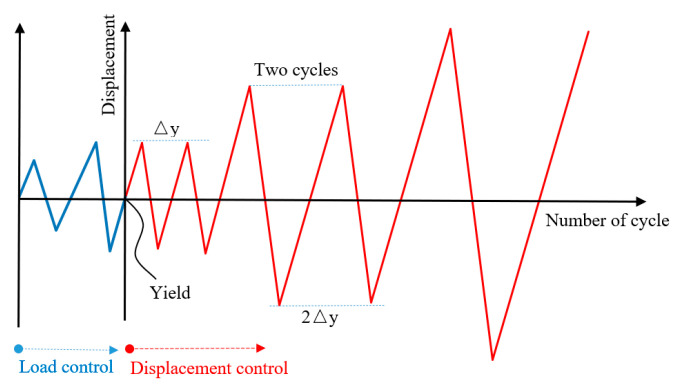
Loading history.

The schematic of the experimental setup and boundary conditions of experimental specimens are shown in Figure 2 and Figure 3. All the pseudo–static tests were conducted using a material testing system (MTS) pseudo–static test system. Before the formal test, a preloading and unloading of the axial compression were first applied to examine the reliability of the test setup and the instrumentations. After the preloading test, a set constant vertical load was applied to the top of the column by a hydraulic jack. Then the electro–hydraulic servo loading system was connected to the actuator for the loading test. Loading was controlled by mixed load and displacement. The loading system is shown in Figure 4. Before yielding, the specimen was loaded by load control, with 75% and 100% of yield load cyclic loading, once per stage. After yielding, the specimen was loaded by displacement control with multiple yield displacements as the step difference, and the displacement amplitude of each step was cycled twice. The pull–down and pull–up were defined as negative and positive, respectively.

## 3. Test Results and Discussion

In order to obtain a deeper understanding of the difference in seismic behavior between conventional and SFRHC joints, the failure modes, hysteretic curve, ductility, and energy dissipation capacity, strength and stiffness degradation were discussed and clarified in detail in this section.

### 3.1. Failure Modes

**Figure 5:**
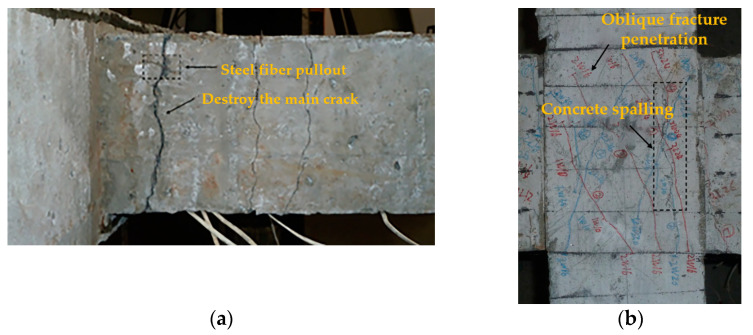
Final failure mode in the joint core area: (**a**) BFF; (**b**) JSF.

**Figure 6:**
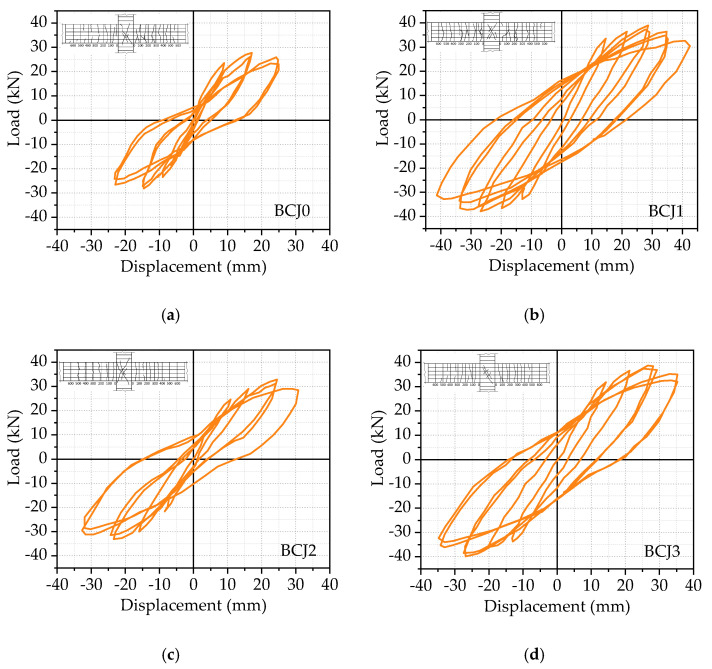

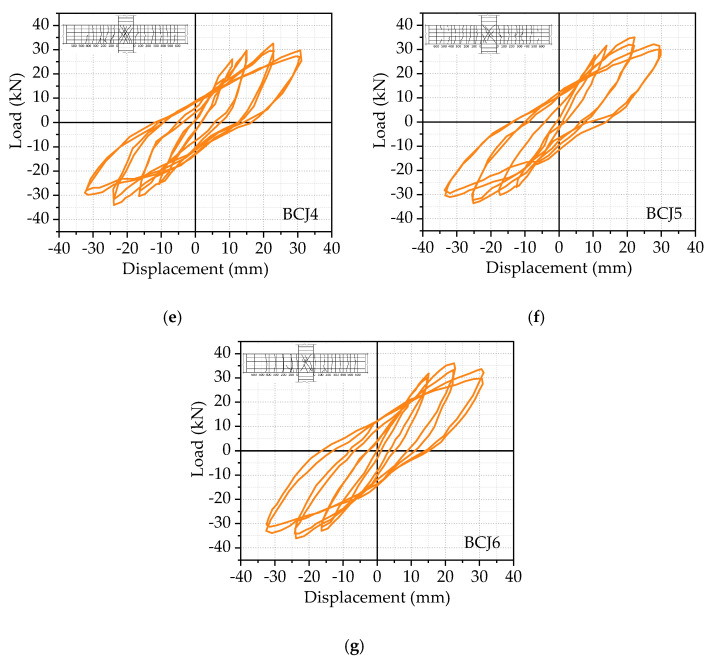
The hysteretic curves and failure modes for the (**a**) BCJ0; (**b**) BCJ1; (**c**) BCJ2; (**d**) BCJ3; (**e**) BCJ4; (**f**) BCJ5; (**g**) BCJ6.

Figure 5 shows the crack diagram of the beam end and core area when the typical specimen is damaged. With the change in stirrup ratio and volume ratio of steel fiber in the core area, the main failure modes of SFRHC beam–column joints can be classified into the following two categories: beam–end–bending failure (BFF) and joint–shear–failure (JSF) in the core area. Figure 6 shows the hysteretic curves and failure modes of these specimens.

**Figure 7:**
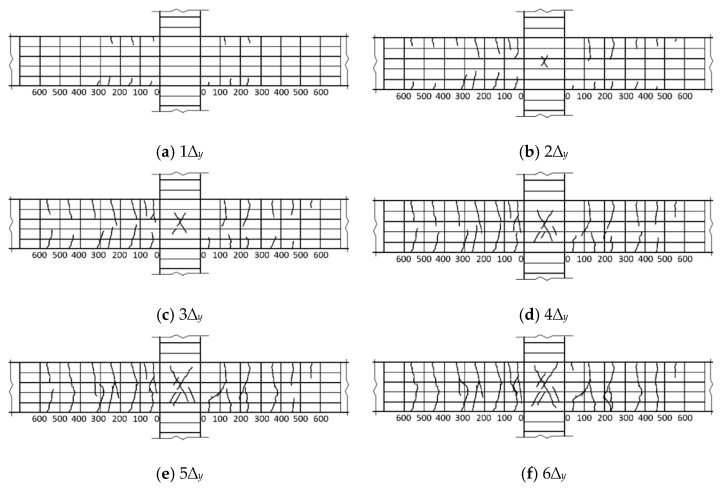
Crack development of specimen BCJ1.

As shown in Figure 5a, The BCJ1 and BCJ3 specimens with large stirrup ratios and volume ratios of steel fiber were bent at the beam ends, and their failure processes were basically the same. Taking the BCJ1 specimen as an example, in the stage of load control, when the load was 12 kN, there was an initial vertical crack with a width of 0.02 mm at the tension zone of 160 and 150 mm from the column edge, respectively, and an initial vertical crack with a width of 0.02 mm at the compression zone, when the tension zone was 180 and 160 mm from the column edge, respectively. With the increase in load, many parallel cracks appeared at the beam end, with an average distance of about 120 mm. In the stage of displacement control, when 1∆*_y_*, the cracks at the end of the beam widened, and new cracks began to appear, but the core area of the BCJs did not crack. When 2∆*_y_* loading, the initial oblique cracks appeared in the core area of BCJs specimens, with a crack width of 0.01 mm and a maximum crack width of 0.6 mm at the beam end. During the 3–4 loading process, the cracks in the core area of BCJs developed slowly, with two small parallel inclined cracks, while the cracks at the beam end developed rapidly, and many new cracks appeared. After 5∆*_y_* loading, the bearing capacity of BCJs decreases, many cracks at the beam end run through the section, and cracks in the core area of BCJs develop slowly. After 6∆*_y_* loading, the bearing capacity of BCJs decreased to 85% of the peak value, and the test ended. The maximum width of cracks at the beam end was 2.6 mm. There were many parallel inclined cracks in the core area of BCJs, which did not penetrate through the core area. The maximum width of cracks was 0.3 mm. See Figure 7 for crack development of the beam–column joint specimen BCJ1.

**Figure 8:**
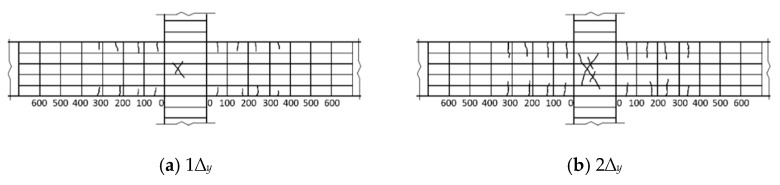

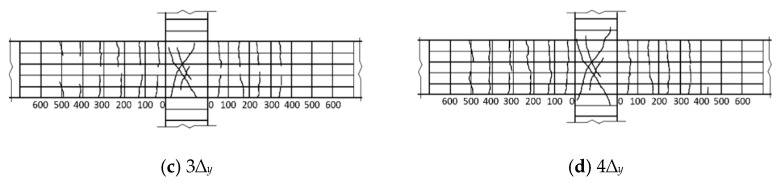
Crack development of specimen BCJ2.

As shown in Figure 5b, it can be found that the BCJs with smaller stirrup ratios or volume ratios of steel fiber in the core area, such as the specimens BCJ0, BCJ2, BCJ4, BCJ5, and BCJ6, were subjected to JSF. The failure process and characteristics of these specimens were as follows: when the imposed load was about 75% of the maximum load, the initial inclined crack with a width of about 0.02 mm appeared in the middle of the joint core area along the approximate diagonal direction. After that, with the increase in load amplitude and cycle times, the inclined cracks spread to two diagonal directions, and the cracks at the beam end developed slowly. When the joint was damaged, the core area of the joint formed a main oblique crack, and a series of small oblique parallel cracks appeared near the middle of the core area. At the same time, most stirrups in the core area had yielded, and local spalling of the concrete cover appeared. The cracks at the beam ends were small and tended to develop stably, and the damage was mainly concentrated in the joint core area. With the change in stirrup ratio and volume ratio of steel fiber, a limited difference of crack development in the core area of joints was observed. Figure 8 shows the crack development process of the specimen BCJ2. From Figure 8, it can be seen that the initial oblique crack appeared later, and the crack developed slowly in the specimen BCJ2. When the cracks were destroyed, the same longer cracks with a slight width were observed, and the small cracks parallel to the main oblique cracks were concentrated, as shown in Figure 6c. In addition, comparing Figure 6a with Figure 6c, it can be seen that the cracking and spalling phenomenon of BCJ1 with a volume ratio of steel fiber of 0.5% was reduced compared to the specimen BCJ0 without steel fiber. The comparison indicated that the crack resistance of steel fiber could significantly improve the failure mode of concrete.

The axial compression ratio of column end on cracks in BCJs is mainly reflected in the cracks in the core area. Initially, inclined cracks appeared in the core area of the BCJ4 specimen with an axial compression ratio of 0.2 at the column end when the load was controlled to 23 kN. The initially inclined cracks appeared in the core area of the BCJ6 specimen with an axial compression ratio of 0.4 at the column end when subjected to cyclic loading twice the yield displacement amplitude. The number of cracks was small during failure, and the cross prominent inclined cracks extended into the column end. Compared to the BCJ1 specimen with a concrete strength of CF60, the initial cracks in the core area of the BCJ3 specimen with a CF80 strength grade appeared later, and the number of cracks was very limited, the oblique cracks were not penetrated, and the width of cracks at the beam end was more petite.

### 3.2. Hysteretic Curve and Skeleton Curve

**Figure 9:**
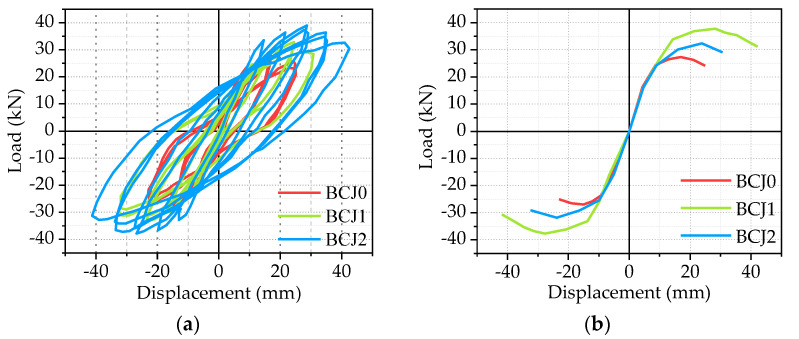
The effect of volume ratio of steel fiber: (**a**) Hysteretic curve; (**b**) Skeleton curve.

**Figure 10:**
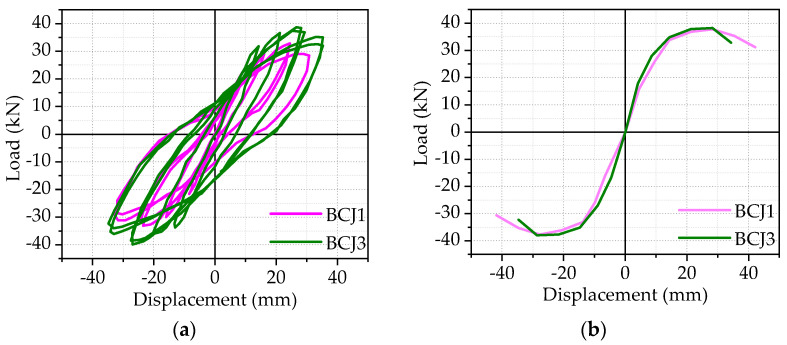
The effect of concrete strengths: (**a**) Hysteretic curve; (**b**) Skeleton curve.

**Figure 11:**
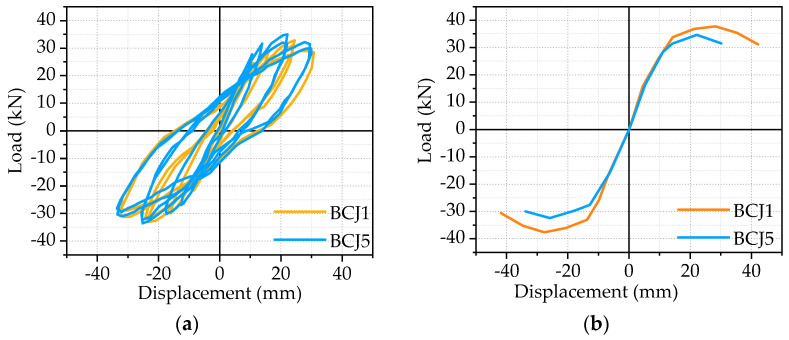
The effect of stirrup ratios in the core area: (**a**) Hysteretic curve; (**b**) Skeleton curve.

**Figure 12:**
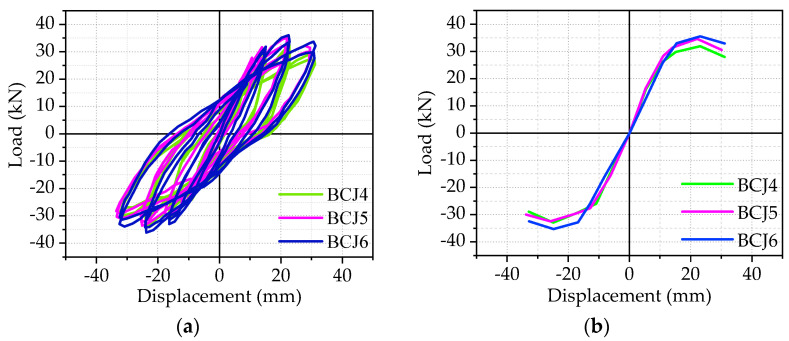
The effect of axial compression ratios: (**a**) Hysteretic curve; (**b**) Skeleton curve.

Hysteric curves from the beam end corresponding to each specimen were shown in the previous subsection, and the skeleton curve of each specimen, which is obtained by connecting the peak points of hysteretic curves of each beam–column joint specimen, will be given in Figure 9, Figure 10, Figure 11 and Figure 12.

- The effect of volume ratios of steel fiber

Figure 9 compares the effect of different volume ratios of steel fiber on the hysteretic curves and skeleton curves, where the volume ratios of steel fiber are 0, 0.5%, and 1%, respectively. At first, it should be noted that the larger the volume ratio of steel fiber is, the plumper the hysteretic loop is, and the stronger the energy dissipation capacity is. The energy dissipation capacity of the BCJ2 specimen is optimal, followed by the BCJ0 and BCJ1 specimens. Compared to the BCJ0 specimen, the load–bearing capacity and the lateral displacement of the BCJ1 specimen were improved by 1.28 and 1.18 times. Meanwhile, in comparison to the BCJ0 specimen, the load–bearing capacity and the lateral displacement of the BCJ2 specimen were improved by 1.15 and 1.14 times. This is mainly due to the application of steel fiber, which can improve the shear strength and tensile strength of the joint core area. The comparisons among the BCJ0, BCJ1, and BCJ2 specimens indicated that steel fiber reinforced concrete can help to improve beam–column joints’ shear strength and deformation capacity. The ultimate displacements of BCJ1 and BCJ2 were 1.42 and 1.75 times of BCJ0, respectively, which proves the excellent deformability of the specimen with steel fiber in the core area. In summary, it can be concluded that the steel fiber can contribute to improving the seismic behavior of BCJs.

- The effect of concrete strengths:

Figure 10 gives the effect of different concrete strengths on the hysteretic curves and skeleton curves, where the concrete strengths are 80.1 and 89.5 Mpa, respectively. Compared to the BCJ1 specimen, the load–bearing capacity of the BCJ3 specimen was improved by 4%, while the corresponding later displacement and failure displacement were reduced by 3% and 13%, with the increase in concrete strength from 80.1 to 89.5 MPa. Therefore, the ultimate bearing capacity increased with the increase in concrete strength, whereas deformability decreased. Meanwhile, increasing concrete strength led to a larger hysteresis loop in shape with a smaller pinching effect.

- The effect of stirrup ratios in the core area:

Figure 11 presents the effect of the transverse reinforcement ratio in the joint core area on the hysteretic curves and skeleton curves, where the stirrup ratio is 0 and 0.6%, respectively. Compared to the BCJ5 specimen, the yield load and corresponding displacement of the BCJ specimen were improved by 6% and 3%, respectively. This is because the shear strength of steel fiber reinforced concrete is high in the early stage, and the influence of the increase in transverse reinforcement in the core area of the joint on the bearing capacity can be neglected. At the later stage of loading, the crack width further increased, the steel fiber reinforced concrete was pulled or pulled out, the transverse reinforcement began to play a role, and the deformation capacity was greatly improved. Therefore, the ultimate displacement of the BCJ1 specimen was 1.29 times that of the BCJ5 specimen.

- The effect of axial compression ratios:

Figure 12 presents the effect of various axial compression ratios in the joint core area on the hysteretic curves and skeleton curves, where the axial compression ratios are 0.2, 0.3, and 0.4, respectively. Compared to the BCJ4 specimen, the load–bearing capacity, corresponding lateral displacement, and limit displacements of BCJ5 and BCJ6 specimens were improved by 1%, 1%, 3%, and 5%, 2%, 5%, respectively, with the increase in axial compression load. In summary, the axial compression load has a slight effect on the bearing capacity and deformation capacity.

### 3.3. Ductility and Equivalent Viscous Damping Ratio

**Figure 13:**
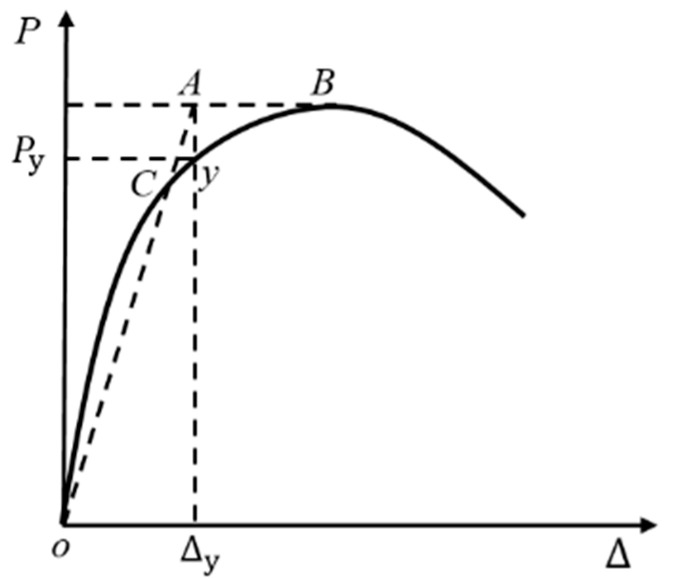
The method to determine yield point.

The ductility of a structure or specimen is usually expressed by ductility coefficient *μ*, which is an important characteristic reflecting the deformability of a structure or specimen. According to the related reference [61], the ductility coefficient is defined as the ratio of the ultimate displacement and yield displacement as follows:

(1) $$\mu =\frac{\Delta_{u}}{\Delta_{y}}$$

where ∆*_u_* is the ultimate displacement when the component load decreased to 85% of the bearing capacity, and ∆*_y_* is the yield displacement of the beam end when the specimen yielded. The energy equivalence method [62] for determining the yield point is plotted in Figure 13. 

It can be seen from Table 3 that, with the increase in volume ratio of steel fiber in the core area of joints, the ductility coefficient of specimens, in general, gradually increased. That is to say, the greater the volume ratio of steel fiber is, the higher the ductility coefficient of SFRHC joints is, and the ductility of the BCJ1 specimen is 2.2 times that of the BCJ1 specimen. Besides, it can be seen from Figure 10 that the reinforced concrete BCJs with stirrups provided a better deformation ability than that of specimens without stirrups, and the ductility coefficient is improved by about 25%. Table 3 and Figure 12 showed that the ductility coefficient of SFRHC joints increases slightly with the increase in axial compression ratio at the column end, indicating that the axial compression ratio of the column end has a slight effect on ductility. Moreover, it can also be seen from Table 3 that with the increase in the strength of SFRHC, the ductility coefficient is decreased by 9%. This is mainly because the workability of concrete decreases with the increase in concrete strength, which leads to easy agglomeration of steel fibers.

**Figure 14:**
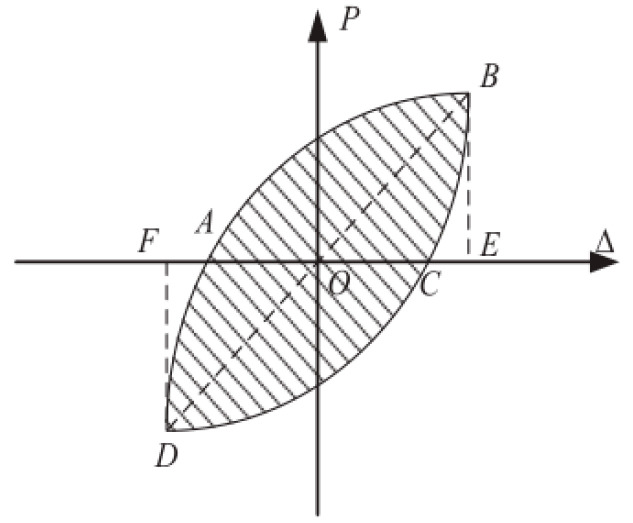
Calculation of the equivalent viscous damping ratio.

**Figure 15:**
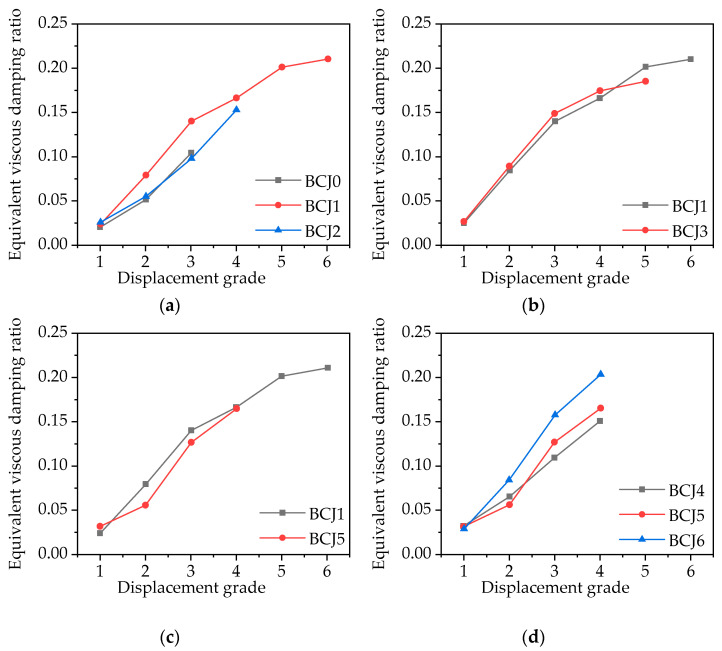
Relationship between the equivalent viscous damping ratios with: (**a**) Different volume ratios of steel fiber; (**b**) Different concrete strength; (**c**) Different stirrup ratios; (**d**) Different axial compression ratios.

Energy dissipation coefficient, work ratio index, and equivalent viscous damping ratio are usually used to quantify the energy dissipation capacity of specimens [63,64,65]. In this paper, the equivalent viscous damping ratio, *h_e_*, is adopted as an index to evaluate the energy dissipation capacity of BCJs specimens. The larger the *h_e_* is, the better the energy dissipation capacity of the specimen is. Figure 14 shows *h_e_* defined and is defined by Equation (2) [65]:

(2) $$h_{e}=\frac{1}{2\pi }\frac{S_{\mathrm{ABCDA}}}{S_{\mathrm{OBE}}+S_{\mathrm{ODF}}}$$

where S_ABCDA_ is the area of each hysteresis loop for a complete loading and unloading; S_OBE_ and S_ODE_ are the triangular areas corresponding to the maximum load and displacement of the hysteresis loop in the forward and reverse directions. The equivalent viscous damping ratio versus the displacement grade (∆/∆*_y_*) of each specimen is provided in Figure 15.

The difference of equivalent viscous damping ratio among all the specimens was very small when longitudinal yielding was not yielding in the initial loading stage. Concrete strength, the axial compression ratio of column end, stirrup ratio, and volume ratio of steel fiber have almost no influence on the energy dissipation capacity of BCJs specimens. After longitudinal yielding, *h_e_* increased with the increase in stirrup ratio, the volume ratio of steel fiber, and axial compression ratio in the later loading stage, which indicates that properly increasing stirrup ratio, the volume ratio of steel fiber, and axial compression ratio are helpful to improve the seismic performance of BCJs specimens. Compared to BCJ1 specimen with concrete strength CF60, the equivalent viscous damping ratio of the BCJ5 specimen with concrete strength CF80 increased by 5% before loading to the peak value, while it decreased by 8% at the final failure. It showed that with the increase in concrete strength, the brittleness of concrete increases, and the energy consumption decreases in the failure stage. The results also showed that the equivalent viscous damping ratios of SFRHC joints BCJ1–BCJ6 were between 0.136 and 0.201, the BCJ0 specimen without steel fiber was 0.085, indicating that SFRHC beam–column joints had a better seismic performance. 

### 3.4. Strength Degradation

**Figure 16:**
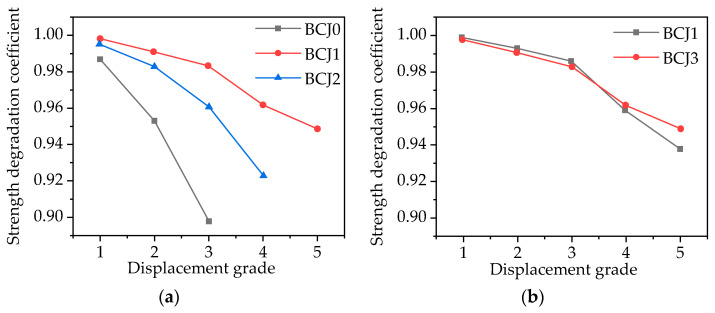

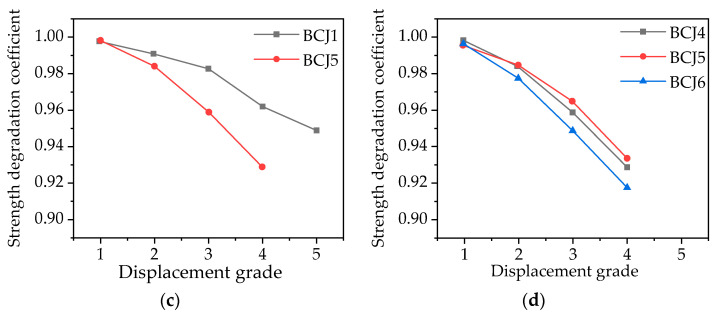
Relationship between strength degradation coefficient and the displacement grade with: (**a**) Different volume ratios of steel fiber; (**b**) Different concrete strength; (**c**) Different stirrup ratios; (**d**) Different axial compression ratios.

The strength degradation characteristic is typically expressed by the strength degradation coefficient (*λ_i_*), which reflects the degree of strength reduction with the increasing cyclic number under the same displacement condition [66]. *λ_i_* is defined in Equation (3):

(3) $$\lambda_{i}=\frac{P_{i,j}}{P_{i,1}}$$

where *P_i_*_,*j*_ represents the peak load value when the displacement grade is *i* (*I* = Δ/Δ*_y_*); *P_i,_*_1_ represents the first cycle peak load value when the displacement grade is *i* (*I* = Δ/Δ*_y_*). *P*_*i*,*j*_ and *P_i,_*_1_ take the average value of peak load under forward and reverse loading. The relationship curve between the strength degradation coefficient and the displacement grade of each specimen is shown in Figure 16.

As shown in Figure 16, under the action of forward and reverse loads, the strength degradation of BCJs specimens was not obvious at the initial stage of loading; then, with the increase in displacement grade, the strength degradation was gradually obvious and showed a steady decreasing trend. Moreover, with the increase in steel fiber volume ratio and transverse reinforcement ratio, the strength degradation tended to slow down. However, concrete strength and axial compression ratio have no obvious influence on strength degradation.

### 3.5. Stiffness Degradation

**Figure 17:**
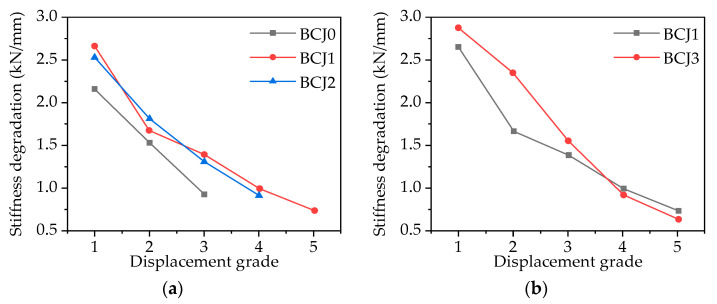

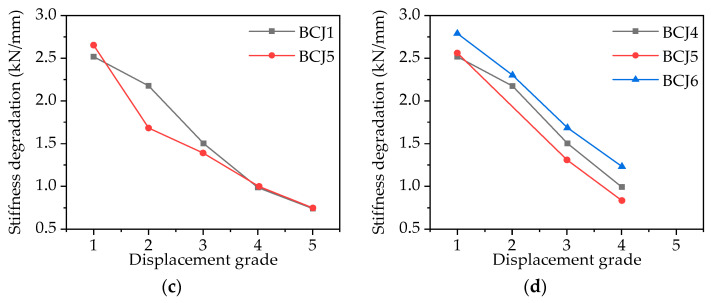
Relationship between stiffness degradation and displacement grade with: (**a**) Different volume ratios of steel fiber; (**b**) Different concrete strengths; (**c**) Different stirrup ratios; (**d**) Different axial compression ratios.

Under the condition of constant displacement, the stiffness of the joint decreases with the increase in cycle times. Stiffness degradation can be measured by the ring stiffness. By analyzing the ring stiffness, the stability and energy dissipation capacity of the hysteretic curve of the joint can be further clarified. The stiffness degradation coefficient (*k_i_*) is defined as follows [66]:

(4) $$k_{i}=\frac{\sum_{j=1}^{n_{k}}P_{i,j}}{\sum_{j=1}^{n_{k}}\Delta_{i,j}}$$

where *P*_*i*,*j*_ represents the peak load value when the displacement grade is *i* (*i* = ∆/∆*_y_*), and the unit is kN; ∆_*i*,*j*_ represents the peak displacement value when the displacement grade is *i* (*i* = ∆/∆*_y_*), and the unit is mm; n represents the number of cycles. Figure 17 shows the relationship between stiffness degradation and the displacement grade of each specimen. The following conclusions can be drawn.

With the increase in displacement, the effective stiffness of the specimens decreased. The decrease in specimen stiffness is usually attributed to joint zone distortion, shear cracking, flexural loss of concrete coating, the slip of reinforcement, and nonlinear deformation of concrete. As shown in Figure 17, the stiffness of SFRHC was obviously higher than that of normal HC, and an increase in approximately 20% was observed for the initial stiffness of the SFRHC than normal HC. This is due to the higher compressive strength and tensile strength of SFRHC compared to normal high–strength concrete. In SFRHC, even with the reduction in transverse reinforcements, the performance was almost the same as that of normal HC, which suggests that the SFRHC specimens provide sufficient shear strength without even a transversal reinforcement in the joint and cause the plastic hinge to be formed in the beam. In Figure 17b, the stiffness of specimen BCJ3 is greater than that of specimen BCJ1 in the early stage of loading, but less than that of specimen BCJ1 in the later stage, which indicates that the brittleness increases with the increase in concrete strength. In addition, it shows that the increase in axial load ratio can reduce stiffness degradation.

## 4. Conclusions

This paper presents an experimental investigation on the seismic behavior of SFRHC beam–column joints by conducting seven tests. The effects of concrete strength, the volume ratio of steel fiber, stirrup ratio in the core area, and axial compression ratio of the column end on the seismic performance of the joint are studied. Based on the experimental results, the main conclusions can be drawn, as follow:

- With the change in axial compression ratio of column end, concrete strength, the stirrup ratio in the core area, and volume ratio of steel fiber, two modes of the joint–shear–failure area exist in the core area and beam–end–bending failure;
- Because of the bridging effect of steel fiber, the initial crack strength and the through crack strength of BCJs can be improved by adding steel fiber into concrete. Moreover, with the increase in volume ratio of steel fiber from 0 to 1%, the ductility and energy consumption of BCJs increase by 45.2% and 120%, respectively, while the degradation of strength and stiffness slows down;
- With the increase in concrete strength from 80.1 to 89.5 Mpa, the ultimate bearing capacity and energy consumption of beam–column joints increase by 4% and 8%, respectively, while the ductility decreases by 9%.
- With the increase in stirrup ratio in the core area from 0 to 0.6%, the ductility and energy consumption of beam–column joints increases by 25% and 20%, respectively;
- The axial compression load has a limited effect on the strength and deformation capacity.

## Figures and Tables

**Table 1:** Detail parameters of the test specimens.

Joint Number	Concrete Strength (Mpa)	Axial Compression Ratio	Volume Ratio of Steel Fiber (%)	Stirrup Ratio (%)	Cubic Compressive Strength (Mpa)	Modulus of Elasticity (Mpa)
BCJ0	C60	0.3	0	0.6	87.4	44,100
BCJ1	CF60	0.3	1.0	0.6	80.1	43,800
BCJ2	CF60	0.3	0.5	0.6	82.1	46,600
BCJ3	CF80	0.3	1.0	0.6	89.5	44,500
BCJ4	CF60	0.2	1.0	0	79.1	43,700
BCJ5	CF60	0.3	1.0	0	81.7	45,300
BCJ6	CF60	0.4	1.0	0	78.1	44,400

Note: C represents the normal concrete; CF represents the steel fiber concrete.

**Table 2:** Mechanical properties of steel bars.

Category	Diameter (mm)	Yield Strength *f_y_* (MPa)	Ultimate Strength *f_u_* (Mpa)	Elastic Modulus (Mpa)	Elongation
HPB235	8	306.9	472.7	209,000	30%
HRB335	16	360.5	594.9	201,000	23%
HRB335	22	418.2	652.1	195,000	27%

**Table 3:** Displacement ductility coefficient and energy dissipation coefficient of specimens.

Joint Number	Yield Load *P_y_* (kN)	Yield Displacement ∆*_y_* (mm)	Ultimate Load *P_u_* (kN)	The Ultimate Displacement ∆*_u_* (mm)	*μ* = ∆*_u_*/∆*_y_*	Equivalent Viscous Damping (*h_e_*)
BCJ0	23.34	9.65	22.02	23.91	2.478	0.085
BCJ1	28.36	11.42	31.22	41.09	3.598	0.187
BCJ2	26.73	10.98	27.43	32.05	2.919	0.136
BCJ3	29.81	11.03	32.39	35.92	3.257	0.201
BCJ4	26.49	10.96	27.68	31	2.828	0.151
BCJ5	26.87	11.05	29.53	31.88	2.885	0.156
BCJ6	27.92	11.14	29.8	32.56	2.923	0.159

## Data Availability

Data are available on request from the authors.

## Nomenclature

BCJs	beam–column joints
SFRHC	steel fiber reinforced high–strength concrete
HC	high–strength concrete
SFRC	steel fiber reinforced concrete
MTS	material testing system
